# Characterisation of the octogenarians presenting to the diagnostic heart failure clinic: SHEAF registry

**DOI:** 10.1136/openhrt-2023-002584

**Published:** 2024-04-24

**Authors:** Luke Thompson, Fiona Carr, Dominic Rogers, Nigel Lewis, Athanasios Charalampopoulos, Graham Fent, Pankaj Garg, Andrew J Swift, Abdallah Al-Mohammad

**Affiliations:** 1 Care of the Elderly, Sheffield Teaching Hospitals NHS Foundation Trust, Sheffield, UK; 2 Department of Cardiology, Sheffield Teaching Hospitals NHS Foundation Trust, Sheffield, UK; 3 Department of Geriatrics, Sheffield Teaching Hospitals NHS Foundation Trust, Sheffield, UK; 4 University of East Anglia, Norwich, Norfolk, UK; 5 Division of Clinical Medicine, The University of Sheffield Faculty of Medicine Dentistry and Health, Sheffield, UK

**Keywords:** HEART FAILURE, Epidemiology, Heart Failure, Diastolic, Heart Failure, Systolic

## Abstract

**Introduction:**

Heart failure (HF) incidence is increasing in older adults with high hospitalisation and mortality rates. Treatment is complicated by side effects and comorbidities. We investigated the clinical characteristics of octogenarians presenting to the HF clinic.

**Methods:**

Data were collected on octogenarians (80–89 years) referred to the HF clinic in two periods. The data included demographics, HF phenotype, comorbidities, symptoms and treatment. We investigate the temporal changes in clinical characteristics using χ^2^ test. We aimed to determine the clinical characteristics which were associated with optimisation of HF pharmacological intervention in the clinic, conducting multivariate regression analysis. Statistical significance is determined at p<0.05.

**Results:**

Data were collected in April 2012 to January 2014 and in June 2021 to December 2022. In this cross-sectional study of temporal data, 571 octogenarians were referred to the clinic in the latter period, in whom the prevalence of HF was 68.48% (391 patients). HF with preserved ejection fraction (HFpEF) was the most common phenotype and increased significantly compared with the first period (46.3% and 29.2%, p<0.001). Frailty, chronic kidney disease and ischaemic heart disease increased significantly versus the first period (p<0.001). During the second period, and following the consultation, of the patients with HF with reduced ejection fraction (HFrEF), 86.4% and 82.7% were on a beta blocker and on an ACE inhibitor/angiotensin receptor blocker/angiotensin receptor-neprilysin inhibitor, respectively. Clinical characteristics associated with further optimisations of HF pharmacological therapy in the HF clinic were: New York Heart Association (NYHA) functional class III and the presence of HFrEF phenotype

**Conclusions:**

With a prevalence of HF at 68% among the octogenarians referred to the HF clinic, HFpEF incidence is rising. The decision to optimise HF pharmacological treatment in octogenarians is driven by NYHA functional class III and the presence of HFrEF phenotype.

**WHAT IS ALREADY KNOWN ON THIS TOPIC**

- There are reports suggesting an increasing prevalence of heart failure with preserved ejection fraction (HFpEF). The magnitude of this increase is to be determined.
- While it is known that octogenarians with heart failure (HF) have a significant number of comorbidities, it is not clear which factors determine the decision to optimise medications in patients with HF.

**WHAT THIS STUDY ADDS**

- There had been a statistically significant rise in the incidence of HFpEF in our HF clinic over the past 10 years.
- The determinants of the decision to optimise medical therapy in patients with HF are New York Heart Association class III and the presence of HF with reduced ejection fraction phenotype.

**HOW THIS STUDY MIGHT AFFECT RESEARCH, PRACTICE OR POLICY**

- Increase the awareness of the magnitude of HFpEF as a problem facing society and concentrating efforts on understanding its pathophysiology to enable future prevention as well as treatment programmes.

## Introduction

Heart failure (HF) is common, affecting nearly 1 million people in the UK. HF is responsible for 5% of all hospital admissions, with an increased incidence in the elderly.^1^ With the UK’s ageing comorbid population, the complexity of HF is increasing, besides the rise of its mortality with age.^2^ Balance needs to be struck between the concerns about the side effects of polypharmacy complicating treatment in the elderly,^3^ and the evidence of benefits of complying with HF treatment guidelines in these patients.^4^

The prevalence of HF with preserved ejection fraction (HFpEF) has increased worldwide.^5^ It is increasingly common in ageing populations^5 6^ and does not have the same evidence base for therapy as that for HF with reduced ejection fraction (HFrEF). In addition, there is an increased recognition of the diagnosis of frailty among patients with HF, which is not just a function of age.^7^ The diagnostic HF clinic in our institution was established in 2012, implementing the National Institute of Health and Clinical Excellence (NICE) guidelines for the diagnosis and treatment of chronic HF, which were updated in 2018.^8^ The European Society of Cardiology guidelines underwent three updates in 2016, 2021 and 2023.^9^

We carried a cross-sectional analysis of the temporal changes over a 10-year period in the clinical characteristics of octogenarians presenting to the diagnostic HF clinic in Sheffield Teaching Hospitals. We also aimed to determine the clinical characteristics that were associated with changes to the pharmacological therapy of the patients attending the clinic.

## Methods

Data were collected from the *S*heffield *HEA*rt *F*ailure (SHEAF) registry of the HF diagnostic clinic at Sheffield Teaching Hospitals.^10^ The population were the octogenarian patients (aged 80–89 years) referred to the clinic with suspected HF and an N-Terminal-pro-Brain Natriuretic Peptide (NTproBNP) >400 ng/L, in two periods: From April 2012 to January 2014, and from June 2021 to December 2022. Data were analysed using Microsoft Excel.

The echocardiograms are carried out by physiologists, the physiologists are accredited by the British Society of Echocardiography (BSE), and where a trainee physiologist does a study, this is reviewed by a senior BSE-accredited physiologist. The minimum data set is gathered where possible. The diagnostic criteria of HFpEF have changed between the two periods of data collection.

We studied the temporal changes in HF phenotype in our cross-sectional study; and further analysed the comorbidities in the two periods of data collection, using χ^2^ test. Statistical significance was determined at p<0.05.

During the second period we compared our performance to the chronic HF quality standards published by NICE in 2022,^11^ particularly Quality statement three calling for adults with chronic HFrEF to receive all appropriate medication at target or optimal tolerated doses. The appropriate medications being: ACE inhibitors (ACEi), beta-blockers, selective aldosterone receptor antagonists (SARA), sodium-glucose co-transporter-2 inhibitors (SGLT2i) and angiotensin receptor-neprilysin inhibitor (ARNi).^8 11^

The analysis included whether these medications were given to patients with HFrEF or not; and the reasons why not given were also analysed. We carried out a multiple regression was done using the stepwise method to analyse the characteristics associated with the decision to optimise pharmacological therapy of the patients with HF. Significance is determined at a p value of <0.05.

## Results

### Demographics

**Figure 1:**
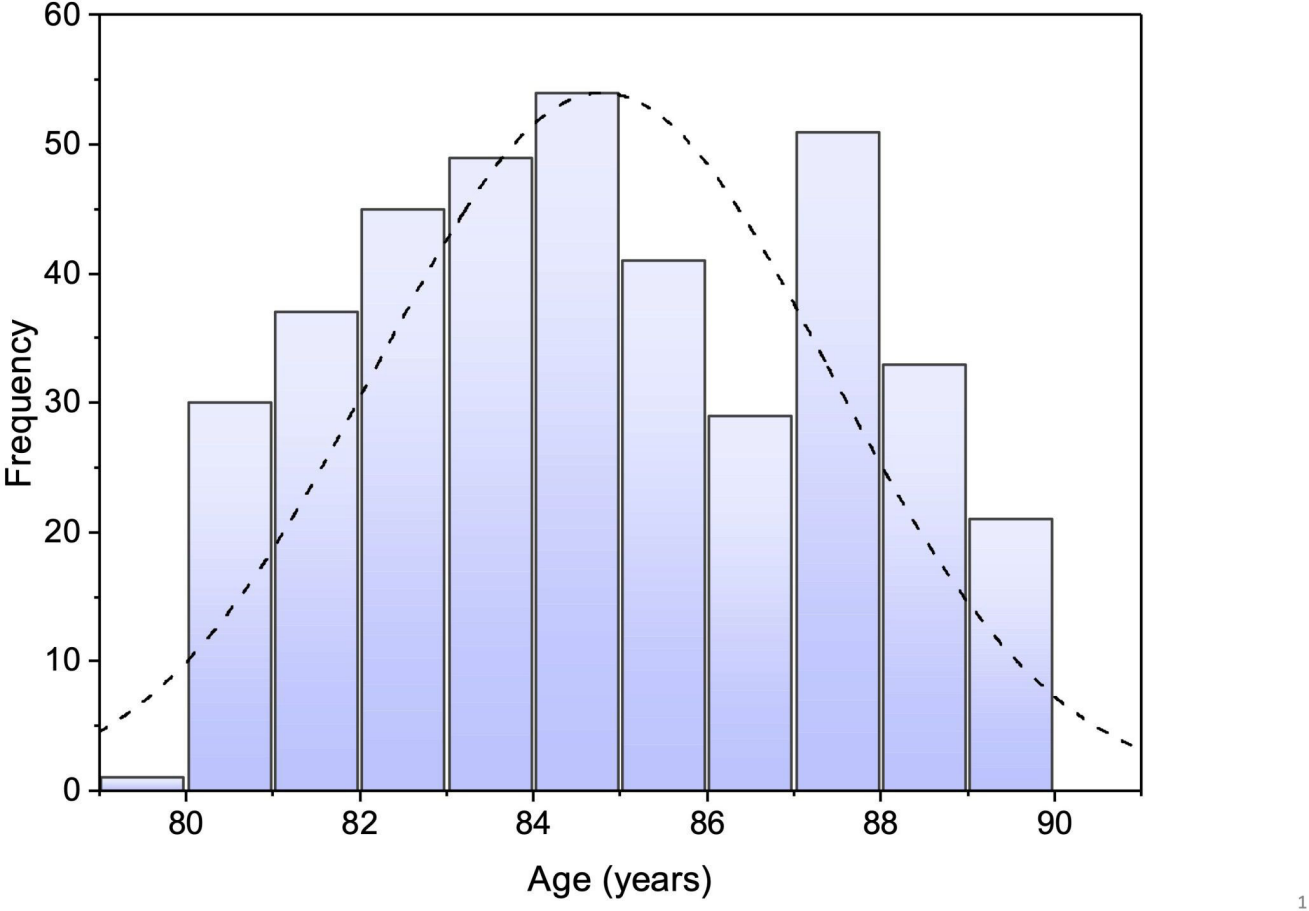
Bar chart showing the distribution of age of octogenarians with heart failure. On the X axis are the age in years, and on the Y axis are the number of patients in each year of age.

The number of octogenarians referred in the second period of data collection was 571 patients. Of these, no evidence of HF was found on echocardiography in 180 patients (32%). Thus, the prevalence of HF among the octogenarians suspected of having HF on the basis of their symptoms and the rise in NTproBNP>400 ng/L,^8^ was 68% (391 patients). The male:female ratio of the octogenarians with HF is 1:1.3. The distribution of age is shown in figure 1.

All patients underwent an ECG and a trans-thoracic echocardiogram (TTE). The distribution of the NTproBNP of the 571 octogenarian patients referred is shown in table 1. However, exceptionally there were 19 patients seen without NTproBNP measurements. Those were 7 patients known to have HF from an inpatient review prior to clinic, 1 was known to have HF from a pre-operative assessment, 8 were known to have HF previously in another hospital and 3 were referred following an ST elevation myocardial infarction. Finally, one patient had an NT-proBNP<400 ng/L, but was known to have HF diagnosed previously at another hospital.

**Table 1:** Distribution of NTproBNP

NTproBNP	Number of patients	Number (%) with confirmed HF
Raised (400–2000)	430	260 (60.5)
High (>2000)	121	111 (91.7)

HF, heart failure; NTproBNP, N-Terminal-pro-Brain Natriuretic Peptide.

### Phenotype of heart failure

**Figure 2:**
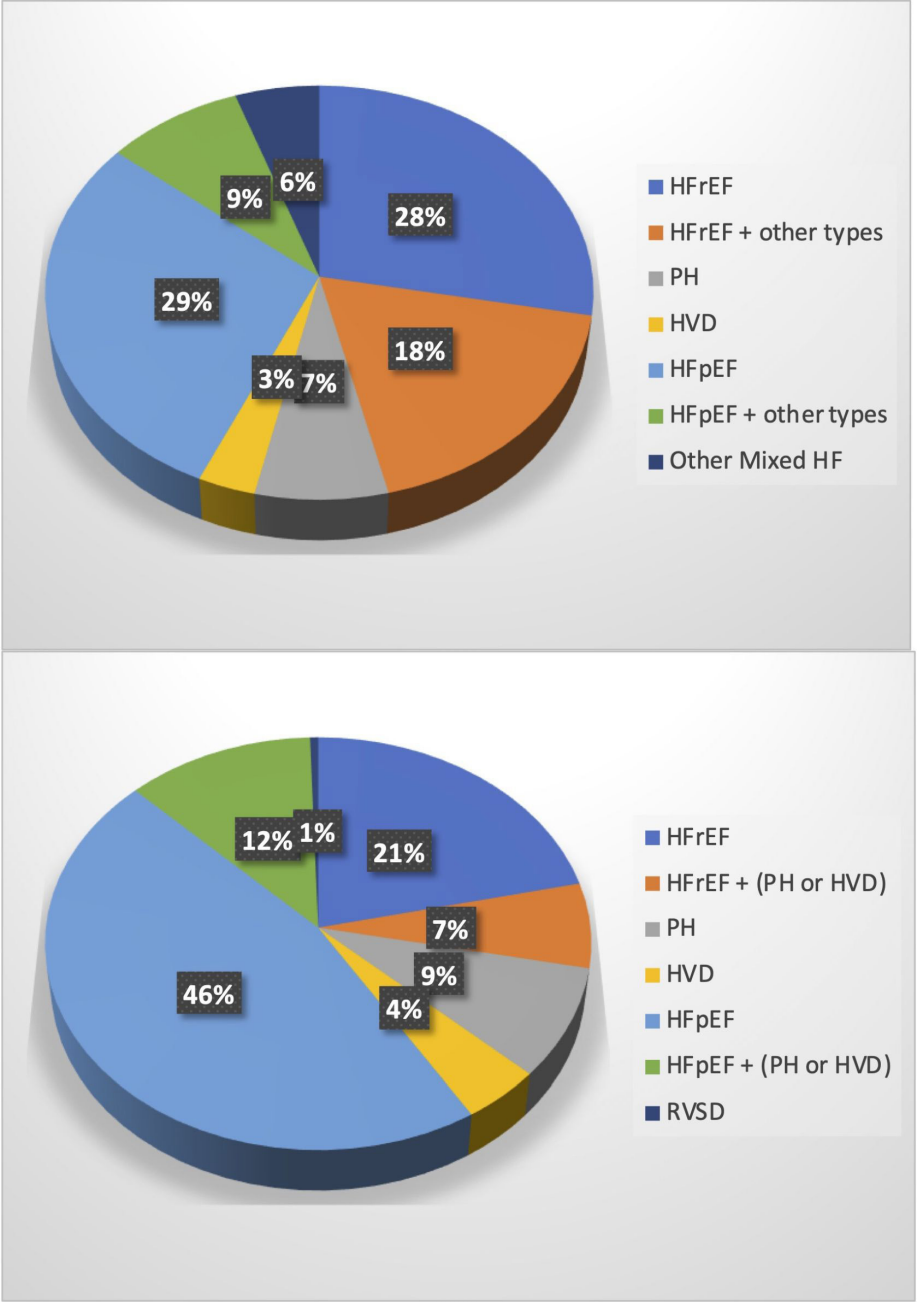
Pie chart showing the distribution of HF phenotype during the first period of data collection (April 2012 to January 2014) (A) and the second period of data collection (June 2021 to December 2022) (B). There has been a statistically significant increase in the diagnosis of HFpEF. HF, heart failure; HFpEF, HF with preserved ejection fraction; HFrEF, HF with reduced ejection fraction; HVD, heart valve disease; PH, pulmonary hypertension; RVSD, right ventricular systolic dysfunction.

The distribution of HF phenotypes during the two periods of data collection is shown in table 2. Pie charts comparing the two data sets are shown in figure 2.

**Table 2:** Distribution of HF phenotypes during the two periods of data collection

Diagnoses in the first period of data collection (April 2012 to January 2014)	No of patients	Diagnoses in the second period of data collection (June 2021 to December 2022)	No of patients	P value
HFrEF	115	HFrEF	84	<0.05
HFrEF with other types	75	HFrEF with other types (PH or HVD)	26	<0.001
PH	30	PH	34	>0.05
HVD	13	HVD	17	>0.05
HFpEF	120	HFpEF	181	<0.001
HFpEF with other types	36	HFpEF with other types (PH or HVD)	47	<0.01
Other mixed heart failure	22			
		Right ventricular systolic dysfunction	2	
Total	411	Total	391	

HF, heart failure; HFpEF, HF with preserved ejection fraction ; HFrEF, HF with reduced ejection fraction ; HVD, heart valve disease; PH, pulmonary hypertension.

During the first period of data collection only 29.2% of patients (120/411) who were proven to have HF had a diagnosis of HFpEF compared with 46.3% (181/391) of patients during the second period of data collection. Thus, there was an almost 59% increase in the proportion of HFpEF as the phenotype during these 10 years. This increase is statistically significant (p<0.001). There was also a significant decrease in the diagnosis of HFrEF singularly or in combination with other types such as heart valve disease (HVD) or pulmonary hypertension (PH).

The distribution considering the total number of patients with HFrEF or HFpEF as the main diagnosis (including those who may have in addition PH or HVD) in the second period (June 2021 to December 2022) is shown in table 3. The patients were classified using the New York Heart Association (NYHA) functional class as shown in table 3. Thus, the majority (85%) had NYHA classes II or III.

**Table 3:** Total number of patients with HFrEF or HFpEF and distribution of patients by NYHA classification; in the second data collection period (June 2021 to December 2022)

Heart failure phenotype	No of patients (% of the total with HF)	NYHA classification	No of patients
HFrEF	110 (28)	I	31
HFpEF	228 (58.3)	II	219
		III	113
		IV	14
		Not coded	14

NYHA, New York Heart Association.

### Comorbidities

The distributions of comorbidities among octogenarians diagnosed with HF during the two periods of data collection are shown in table 4. There has been a statistically significant increase in the diagnoses of frailty, chronic kidney disease (CKD) and ischaemic heart disease (IHD) (p<0.001).

**Table 4:** Distribution of comorbidities during the two periods of data collection (April 2012 to January 2014) and (June 2021 to December 2022)

Comorbidity	No. of patients (% of the total with HF) April 2012 to January 2014	No. of patients (% of the total with HF) June 2021 to December 2022	P value
Hypertension	292 (71)	333 (85.1)	<0.001
Ischaemic heart disease	66 (16)	116 (29.7)	<0.001
Chronic kidney disease	218 (53)	330 (84.3)	<0.001
Cancer	38 (9.2)		
Stroke	90 (21.9)	40 (10.2)	<0.001
Diabetes mellitus	72 (17.5)	78 (19.9)	>0.05
Respiratory	185 (45)		
Arrhythmias	110 (26.8)		
Chronic obstructive pulmonary disease		74 (18.9)	
Interstitial lung disease		21 (5.3)	
Peripheral vascular disease	18 (4.4)	20 (5.1)	>0.05
Cognitive impairment	35 (8.5)	51 (13)	<0.05
Frailty	15 (3.6)	159 (38.6)	<0.001
Pulmonary emboli		7 (1.8)	
Hypercholesterolaemia		128 (32.7)	
Ex-smoker		157 (40.2)	
Haematological	38 (9.2)		

Where a comorbidity is estimated during only one period then a blank space is left in the respective year’s column.

HF, heart failure; HFpEF, HF with preserved ejection fraction ; HFrEF, HF with reduced ejection fraction .

### Optimisation of pharmacological therapy

Of 391 octogenarian patients with HF during the second period of data collection (June 2021 to December 2022), 290 patients (74%) had changes made to optimise their treatment. These changes were either made in clinic or suggested for either the general practitioner or the HF specialist nurses to implement. Changes included starting new medications, up-titrating medications or changing other medications. Further analysis was undertaken of the 110 octogenarian patients with HFrEF, and this is summarised in table 5.

**Table 5:** Number of octogenarian patients with HFrEF on different medications after the clinic consultation

Medication	No. of octogenarian patients on the medication after clinic consultation (% of total octogenarian patients with HFrEF)
Beta-blockers	95 (86.4)
ACEi/ARB/ARNi	91 (82.7)
SARA	57 (51.8)
SGLT2i	12 (10.9)

ACEi, ACE inhibitors; ARB, angiotensin receptor blocker; ARNi, angiotensin receptor-neprilysin inhibitor; HFrEF, heart failure with reduced ejection fraction ; SARA, selective aldosterone receptor antagonists; SGLT2i, sodium-glucose co-transporter 2 inhibitor.

9 of the 15 patients not on a beta blocker had a listed justification such as intolerance or a contraindication. Similarly, 14 of the 19 patients not on an ACEi, an angiotensin receptor blocker (ARB) or ARNi were justified as contraindicated because of renal dysfunction.

#### The clinical characteristic associated with optimisation of pharmacological therapy

**Figure 3:**
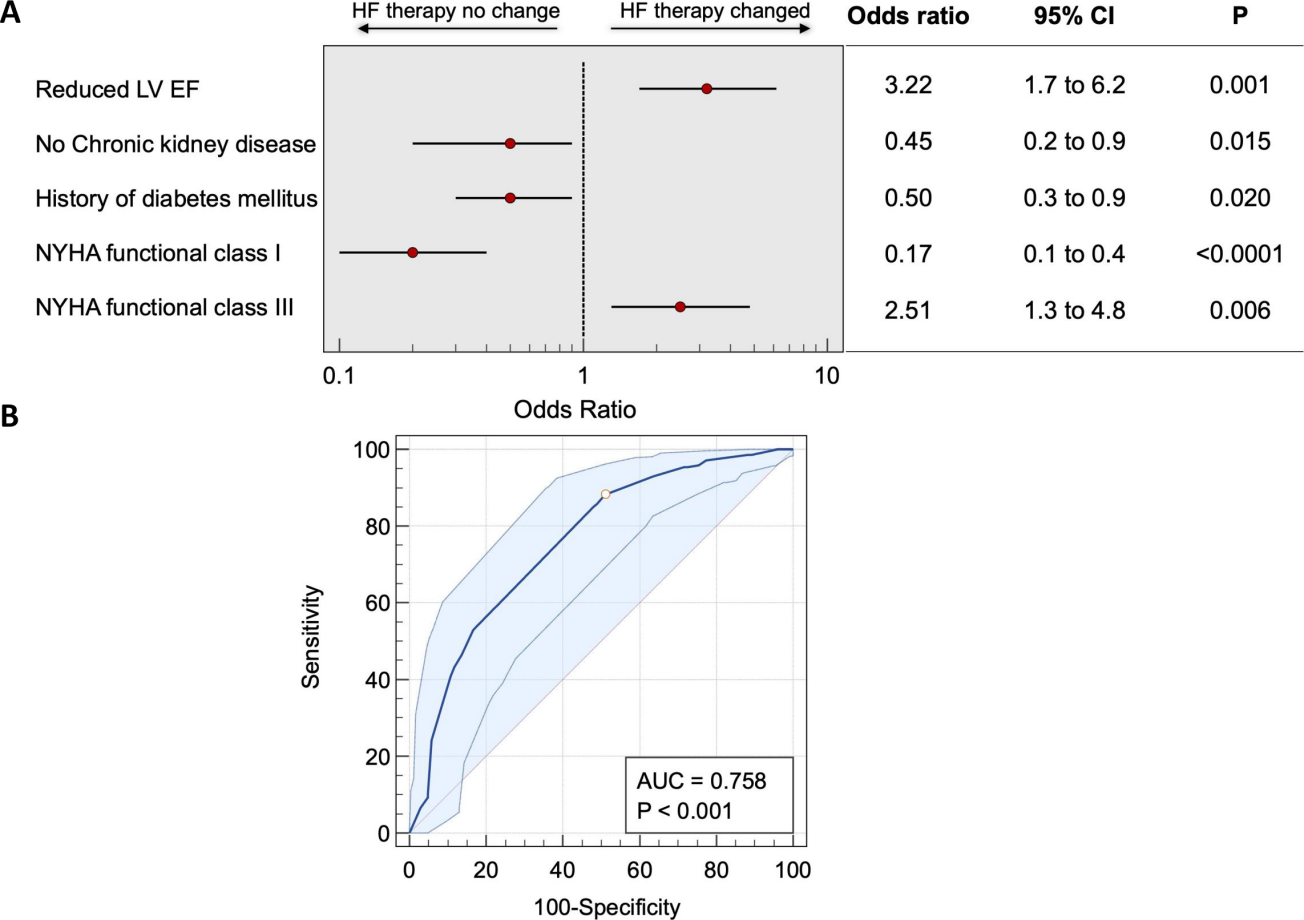
The multiple regression was done using the stepwise method. Non-significant data is not presented as it is redundant information. Increasing symptoms represented by NYHA functional class III and the presence of the HF with reduced ejection fraction phenotype are the factors that accurately predict which patient gets optimisation of the HF therapy. (A) The forest plots from the multivariate analyses of the significant variables only. (B) ROC of how these variables were able to predict change in heart failure therapy. AUC, area under the curve; HF, heart failure; LVEF, left ventricular ejection fraction; NYHA, New York Heart Association; ROC, receiver operating characteristic curve.

Multivariate regression analysis of the clinical characteristics that were associated with optimisation of pharmacological therapy for octogenarian patients with HF was undertaken. The analysis tested whether age, NYHA class, comorbidities (such as hypertension, IHD, CKD, hypercholesterolaemia, stroke, diabetes mellitus, peripheral vascular disease, chronic obstructive pulmonary disease, being a current or an ex-smoker, interstitial lung disease, pulmonary emboli, frailty or cognitive impairment) or HF phenotype (HFpEF or HFrEF) affected whether changes were made to pharmacological therapy. Several factors were rejected in the stepwise regression model, and the remaining significant factors are shown in figure 3. Essentially, two major factors were associated with the chance of optimising the pharmacological therapy: the presence of HFrEF phenotype and the presence of significant symptoms as defined by NYHA class III (figure 3).

## Discussion

HF causes significant morbidity and mortality, and has a growing prevalence in the UK. Patients’ age and comorbidity burden at first presentation have increased over time, further increasing the impact of patients with HF on health services as their management can be more complex.^1^ Data regarding the treatment of HF in the elderly is limited. The data from trials on elderly patients, which are mainly from subgroup analyses suggest that current clinical guidelines should be followed.^4 12^ Comorbidities are more common in the elderly and may play a significant role in treatment choices and in patients’ compliance. Thus, these comorbidities will have an exaggerated impact on prognosis by impeding drug therapy.^13^ Polypharmacy is also more likely in this population, increasing the likelihood of drug interactions and adverse effects.

In our cross-sectional analysis of the octogenarian patients presenting to the HF clinic over two periods (April 2012 to January 2014) and (June 2021 to December 2022); demonstrated HF was found in 411 and 391 octogenarian patients, respectively. During the latter of the two periods, 68.48% of the octogenarians referred to the clinic had HF. Interestingly, the predictability of HF based on the octogenarian patient’s NTproBNP was 60.5% of those with NTproBNP 400–2000 ng/L, and 91.7% of those with NTproBNP>2000 ng/L. The respective chances of HF diagnosis being made in our large cohort from the same SHEAF registry including all age groups were similar at 64% and 92%, respectively.^14^

The diagnosis of HF is a clinical one. However, not all patients with breathlessness or peripheral oedema with raised NRproBNP have HF. Cardiac imaging confirms or refutes the presence of cardiac dysfunction responsible for the syndrome. TTE is the most commonly used imaging technique in the diagnosis of HF. Those who do not have left ventricular systolic dysfunction, right ventricular systolic dysfunction or pulmonary hypertension need to have abnormal markers of diastolic left ventricular function to establish the diagnosis of HFpEF. The latter follows the criteria set by the European Society of Cardiology (ESC). We follow the most recent iteration of the ESC criteria. Not many octogenarian patients in our cohort had undergone an assessment of global longitudinal strain or left atrial strain.

Those who do not fulfil the diagnostic echocardiographic criteria for HF,^10^ may undergo further non-invasive or invasive cardiac investigations where the suspicion of HF remains high, or be referred for other non-cardiac investigations as may be appropriate. We do not have from the SHEAF registry all the alternative diagnoses of the patients who did not have HF. We have previously characterised the latter group of patients in our SHEAF registry (not just the octogenarians).^10^ While we know from the literature that HFpEF prevalence is increasing,^5^ it was interesting in our cross-sectional study that there was statistically significant, almost 59% rise in the prevalence of HFpEF over a 10-year period. However, there is a possible confounder that we have to acknowledge here: the criteria of diagnosing HFpEF changed between the two periods of data collection. In particular the E:e’ threshold for making the diagnosis was lowered, and there was an addition to the criteria acknowledging the potential for left ventricular hypertrophy or a pulmonary artery pressure measured by the tricuspid regurgitation jet above 35 mm Hg being sufficient along with the dilatation of the left atrium to make the diagnosis of HFpEF even when the E:e’ is not raised. We remain uncertain how much of the 59% rise in the incidence of HFpEF is reflecting this change of the diagnostic criteria and how much reflects a genuine rise in the incidence of a pathophysiological phenomenon due to epidemiological factors driven by the rising number of the elderly comorbid population with time.

We found an increased prevalence of CKD, IHD and frailty among these octogenarians in comparison with those diagnosed with HF between 2012 and 2014. Our findings are similar to those of other studies. Population-based studies in the UK show that over time the number of patients being diagnosed with HF is increasing. They also showed the number of comorbidities at presentation is increasing along with the prevalence of individual comorbidities, such as: CKD, and IHD.^1^ Studies of electronic health records in England show that prevalence of frailty is increasing.^15^ Meta-analyses of HF studies show a high prevalence of frailty and that those with frailty have an increased risk of hospitalisation and mortality.^7 16^

Among our patients found to have HFrEF, 86.4% and 82.7% were treated with a beta blocker or with one of ACEi/ARB/ARNi, respectively. On the other hand, only 51.8% of the octogenarian patients with HFrEF were on SARA. While none of these percentages significantly differ from the average reported in the UK National HF audit, which looks at the patients hospitalised with HF (unlike our outpatients cohort), one really would hope that the rate of prescribing SARA for this indication does improve with time.

Another important finding from our investigation in this study was the determination of the clinical characteristics associated with the clinician’s decision to make therapeutic changes for the octogenarian patients. We are aware that octogenarian patients are particularly affected by larger than average number of comorbidities, which could hinder optimisation of medical therapy for HF, particularly in the presence of HFrEF as a diagnosis. In our cohort collected in the second period, age was not a strong enough factor to even feature in the multivariate regression analysis. However, the factors that did affect those decisions were: the presence of significant HF symptoms as described by NYHA functional class III and the diagnosis of HFrEF. The fact that patient’s symptoms are one of the significant drivers to make changes in the management is not only reassuring but also plausible.

The low uptake of SGLT2i among the group is related to the timing of these agents being licensed by NICE for the treatment of patients with HFrEF.^17 18^ As for their use in HFpEF, dapagliflozin was not recommended by NICE until June 2023,^19^ while empagliflozin use in HFpEF was only recommended by NICE in October 2023.^20^

### Limitations

We recognise that our study suffers from the limitations of the retrospective nature of our cross-sectional study, which is observational by definition. In addition, the classification of the comorbidities was not entirely unified between the two periods of data collection. We elected not to alter the row data provided during the first period of data collection to achieve harmonisation of the classification of the comorbidities. The list of comorbidities may not be comprehensive. Depression is notably absent from the list of comorbidities. In addition, some of these comorbidities were cited in a binary fashion even though they vary in severity and impact as defined in the Clinical Frailty Score.

We are intrigued by the magnitude of the rise of the incidence of HFpEF over the 10-year period, but we do acknowledge that we have not provided an assessment of the potential impact of the changes to the diagnostic criteria of HFpEF on this rise.

In our study we did not compare the octogenarians as a group to a younger population to study the potential impact in general on the management decisions.

## Conclusions

Treatment decisions need to be individualised particularly when patients with HF are elderly with complex comorbidities. The concerns about poly-pharmacy, side effects and frailty are appropriate in this population. However, these concerns are not acceptable excuses for treatment complacency as many of these octogenarians stand to benefit from the disease modifying therapy particularly those available for patients with HFrEF; and more recently with the use of SGLT2i in HFpEF.^19 20^ The significant reductions in symptoms and in the risk of hospitalisation afforded by these therapeutics are worthy of every effort made by the healthcare professions to provide patients with.

## Data Availability

All data relevant to the study are included in the article or uploaded as supplementary information.
